# Physical Activity and Quality of Life in Cancer Survivors: A Meta-Synthesis of Qualitative Research

**DOI:** 10.3390/cancers9050053

**Published:** 2017-05-20

**Authors:** Shaunna Burke, Amanda Wurz, Andrew Bradshaw, Stephanie Saunders, Malcolm A. West, Jennifer Brunet

**Affiliations:** 1 School of Biomedical Sciences, Faculty of Biological Sciences, University of Leeds, Leeds LS29JT, UK; sp12a2b@leeds.ac.uk; 2 School of Human Kinetics, Faculty of Health Sciences, University of Ottawa, Ottawa ON K1N6N5, Canada; amandawurz@uottawa.ca (A.W.); ssaun028@uottawa.ca (S.S.); jennifer.brunet@uoottawa.ca (J.B.); 3 Integrative Physiology and Critical Illness Group, Clinical and Experimental Sciences, Faculty of Medicine, University of Southampton, Southampton SO17 1BJ, UK; m.west@soton.ac.uk; 4 Critical Care Research Area, Southampton NIHR Respiratory Biomedical Research Unit, Southampton SO16 6YD, UK; 5 Anaesthesia and Critical Care Research Unit, University Hospital Southampton NHS Foundation Trust, Southampton SO16 6YD, UK; 6 Academic Unit of Cancer Sciences, University Hospital Southampton NHS Foundation Trust, Southampton SO16 6YD, UK; 7 Institut du Savoir de l’Hôpital Montfort (IRHM), Hôpital Montfort, Ottawa ON K1K 0T2, Canada; 8 Cancer Therapeutic Program, Ottawa Hospital Research Institute (OHRI), Ottawa ON K1H 8L6, Canada

**Keywords:** quality of life, physical activity, cancer, qualitative research

## Abstract

Qualitative research on the impact of physical activity on quality of life (QoL) in adults diagnosed with cancer is accumulating. However, the field of physical activity and cancer survivorship lack a synthesis of this research to reliably understand the implications for future research and practice. The aim of this meta-synthesis was to identify, appraise, and synthesize qualitative research on cancer survivors’ perspectives of the impact of physical activity on their QoL. Seven electronic databases were searched for original studies published in English, and reference lists of relevant studies were hand-searched to identify additional studies. Forty studies met eligibility criteria and were included in this meta-synthesis. Study characteristics and major findings were extracted, and findings were summarized, compared, and synthesized. Themes identified in this review revealed that physical activity positively impacted four dimensions of cancer survivors’ QoL: *physical* (e.g., managing the physical consequences of cancer and its treatment), *psychological* (e.g., evoking positive self-perceptions), *social* (e.g., feeling understood by others), and *spiritual* (e.g., redefining life purpose). This meta-synthesis corroborates conclusions from reviews of quantitative research and illustrates that physical activity can be used to improve QoL in adult cancer survivors, regardless of diagnosis (i.e., stage, cancer type) and treatment status. It also provides detailed insight into specific aspects within each dimension of QoL impacted by physical activity from cancer survivors’ perspectives, which is important for understanding the meaning and utility of physical activity for them. However, more research is needed to further develop the qualitative evidence base in order to better understand how physical activity impacts on QoL experiences in men, young adults, and adults diagnosed with less common types of cancer at different points along cancer trajectory (i.e., diagnosis, treatment, post-treatment, palliation).

## 1. Introduction

Worldwide, approximately 14.1 million people are diagnosed with cancer each year [1]. Advances in research, early detection, and treatment options have improved survival rates, with an estimated 32.6 million adults expected to live at least 5-years post-diagnosis [1]. Together, this has given rise to a growing population of cancer survivors (i.e., individuals with cancer from the point of diagnosis onwards [2]). Many cancer survivors report adverse physical (e.g., persistent fatigue, pain, weight gain, decreased muscle capacity, reduced immune function) and psychosocial side effects (e.g., psychological distress, anxiety, social isolation, fear of recurrence) that can persist for months or years following treatment [3,4,5,6]. In turn, these side effects can have a profound detrimental impact on survivors’ quality of life (QoL) [7].

QoL is a multidimensional construct that reflects a person’s subjective evaluation of their well-being and functioning across multiple life domains [8], each of which should be targeted through cancer support services and resources because QoL is considered an important outcome measure in healthcare [9]. According to Ferrans [10], there are five key dimensions of QoL: (1) physical; (2) functional; (3) psychological/emotional; (4) social; and (5) spiritual. A considerable number of studies focusing on QoL have been conducted over the last few decades to determine if physical activity can improve cancer survivors’ evaluation of their QoL. Reviews of quantitative research show that physical activity can improve physical, psychological/emotional, and social functioning, reduce treatment-related side effects (e.g., pain, fatigue, nausea), and enhance general life satisfaction among cancer survivors [11,12,13,14,15,16,17,18]. For example, Albrecht and Taylor [14] found positive associations between physical activity and QoL among patients with advanced-stage cancer in seven of the nine studies they reviewed. Specifically, they reported that physical activity was associated with lower levels of anxiety, stress, depression, and cancer-related symptoms (e.g., pain, fatigue, shortness of breath, constipation, insomnia). Similarly, Mishra et al. [17] reviewed 40 studies and concluded that physical activity had beneficial effects on cancer survivors’ general QoL (standardized mean difference (SMD) = 0.99) and specific aspects of QoL, including self-esteem (mean differences = 2.70–4.50), emotional well-being (SMD = 0.33), sexuality (SMD = 0.40), sleep disturbance (SMD = −0.46), social functioning (SMD = 0.45–0.49), anxiety (SMD = −0.26), fatigue (SMD = −0.82–−0.55), and pain (SMD = −0.29). McNeely [16] also reported that physical activity led to significant improvements in cancer survivors’ QoL (weighted mean differences = 4.58–6.62) in their review of 136 studies. Whilst previous syntheses of existing quantitative research have provided consistent and ample scientific evidence to suggest that physical activity facilitates QoL among adult cancer survivors, there is considerable heterogeneity across studies. Sources of variation observed in the studies reviewed include different measures, sample sizes, types of participants, measurement time intervals, and intervention elements (e.g., length, frequency, duration, intensity). This lack of uniformity across studies may explain, at least in part, the observed heterogeneity. However, it can also be argued that quantitative methods used to assess QoL fail to cover all aspects of QoL that cancer survivors feel have been impacted by physical activity. This oversight is likely to underestimate the impact of physical activity on QoL by neglecting to capture the range, depth, and complexity of survivors’ QoL experiences.

As a result, the use of diverse methodologies to investigate the impact of physical activity on QoL in adult cancer survivors has grown. In particular, qualitative methods are increasingly being used to collect comprehensive data on cancer survivors’ personal perspectives of QoL, ultimately allowing for a better understanding of the meaning and utility of physical activity during cancer survivorship [19,20,21]. However, evidence from a single qualitative study on its own is not as persuasive to inform practice as evidence from a cumulative body of qualitative research that shows consistent results. Despite this, efforts to synthesize and integrate findings from existing studies using qualitative methods to investigate the impact of physical activity on QoL in cancer survivors are lacking. Therefore, this review aimed to systematically investigate the extent to which studies that used qualitative methods provide evidence for the impact of physical activity on QoL from cancer survivors’ perspective.

### The Importance of Qualitative Research and Methods to Summarize Findings

Qualitative research involving flexible research designs, varied methods of data collection (e.g., semi-structured interviews, focus groups, observations), and subjectivist epistemologies allows for an in-depth exploration of cancer survivors’ perspectives of the impact of physical activity on their QoL and provides insight into what is meaningful for them [22]. This body of work is important as it can be integrated within the larger body of knowledge on this topic, which is mostly derived from quantitative research. A review of qualitative research can provide detailed information regarding how cancer survivors view physical activity as contributing to their physical, functional, psychological/emotional, social, and spiritual QoL. For this reason, it is necessary to identify and synthesize qualitative research to identify research gaps, aid planning of future research, and inform practice.

Qualitative meta-synthesis is one approach that uses rigorous methods to identify, analyze, and critically appraise the findings of multiple studies related to a specific research topic in order to generate a holistic understanding of the phenomenon under investigation [23,24]. The contribution of meta-syntheses for expanding the evidence base for practice in health-related fields is increasing and is crucial for the development, evaluation, and implementation of interventions [24]. Thus, the objective of this review was to conduct a meta-synthesis by analyzing, synthesizing, and interpreting qualitative findings on cancer survivors’ perspectives of how physical activity impacts their QoL in order to present an overall view of the aspects of QoL that cancer survivors feel are impacted.

## 2. Methods

When undertaking this meta-synthesis, recommendations outlined by Paterson et al. [25] were followed. The first step involved a systematic search to identify relevant studies presenting qualitative findings. Seven electronic databases were searched: Medical Literature Analysis and Retrieval System Online (MEDLINE), Cochrane Central Register of Controlled Trials (CENTRAL), Cumulative Index to Nursing and Allied Health Literature (CINAHL), PsycINFO, Scopus, SPORTDiscus, and Web of Science. With the help of a university librarian (Karine Fournier), a sensitive search strategy was developed drawing on keywords that have been used in published reviews e.g., [17]. Medical Subject Headings (MeSH) terms and keywords that were used covered: the target population (i.e., cancer patients/survivors), intervention (i.e., physical activity), outcome (i.e., QoL), and methods (i.e., qualitative). The search strategy was pilot tested and finalized in MEDLINE (see Appendix Table A1 for the final MEDLINE search strategy) before being translated for use in the six other databases. The electronic search took place in October 2016, after which all identified citations were imported to a reference management software (EndNote) and duplicates were removed. At this point, two authors (Andrew Bradshaw, Stephanie Saunders) independently screened studies through two stages: (1) titles and abstracts; and (2) full-texts. At each step of the screening process, studies were excluded if they did not meet the inclusion and exclusion criteria described below. In instances of uncertainty or disagreement, two additional authors (Shaunna Burke, Amanda Wurz) were available for further discussion. For studies with insufficient details to assess eligibility, further details were sought from the corresponding author of each study in order to determine their relevance to this review. Concurrently, reference lists of key articles and reviews retrieved during the database searches were hand-searched to ensure all relevant studies were identified.

Studies were included in this meta-synthesis if they: (1) were conducted with samples comprised of adults (≥18 years) diagnosed with cancer, regardless of type of cancer, stage of the disease, and point along cancer trajectory (e.g., diagnosis, treatment, post-treatment, palliation); (2) used qualitative methods to collect data (e.g., interviews, focus groups, observations); (3) had participants engage in physical activity of any type and intensity; (4) were original research published in English language in a peer-reviewed journal; and (5) presented qualitative data on at least one domain of QoL as a primary, secondary, or tertiary outcome. No restriction was placed on year of publication. Mixed methods studies in which qualitative findings were not presented were excluded. Moreover, studies in which participants engaged in a single session of physical activity and/or in which participants received an intervention targeting multiple health behaviors (e.g., physical activity and nutrition) were excluded.

### 2.1. Quality Assessment

Three authors (Andrew Bradshaw, Stephanie Saunders, Shaunna Burke) independently assessed the quality of the included studies using criteria consistent with the ontology, epistemology, methodology, and methods of each study. This approach followed the relativist perspective outlined by Sparkes and Smith [26]. The three authors completed this task by appraising the trustworthiness, theoretical considerations, and practical considerations of each study using Garside’s [27] criteria alongside Williams et al.’s [28] quality appraisal questions. However, to compensate for differences in the methodological approaches and philosophical assumptions underlying each study, additional and alternative criteria were used where appropriate [26]. The final grading of the methodological quality of each study was reported as “high”, “medium”, or “low” (see Table 1).

Excluding studies from a meta-synthesis on the basis of quality is debated amongst researchers [25,29]. Given the general lack of consensus concerning quality in qualitative research, many have argued against excluding studies on this basis. For example, Walsh and Downe [24] stated that the contribution individual studies make to knowledge is more important than their rigour. Thus, studies were not excluded from this review based on quality as all were likely to be relevant to the research objective and contribute to the overall understanding of cancer survivors’ perspective of the impact of physical activity on their QoL.

### 2.2. Data Abstraction

For each study included in this review, the following data were abstracted by two authors (Andrew Bradshaw, Stephanie Saunders) independently using a template for collecting data (see Appendix Table A2): country of origin, objective(s), sample characteristics (i.e., age, sex, type of cancer, stage of the disease, point along the cancer trajectory), physical activity intervention characteristics, methodology, methods, conceptual/theoretical approaches, and key qualitative findings. A third author (Shaunna Burke) then verified the accuracy of the data extracted and recorded.

### 2.3. Data Analysis

Data analysis involved three main analytical steps: meta-data analysis, meta-method analysis, and meta-theory analysis [25]. The meta-data analysis was guided by a framework analysis approach [30]. This approach is well-suited for meta-syntheses in light of the diverse methodologies used across studies. Framework analysis involved identifying common themes and subthemes across studies. First, one author (Shaunna Burke) familiarized herself with the textual data to become aware of the key findings presented in each study. Second, the same author (Shaunna Burke) coded the data using a deductive approach whereby connections to the broad dimensions of QoL were made. Third, similar codes were grouped together into categories within each dimension of QoL to form a working analytic framework. Fourth, several iterations of the analytical framework were created until no additional codes emerged. Fifth, each code was assigned a number and then four authors (Shaunna Burke, Amanda Wurz, Andrew Bradshaw, Stephanie Saunders) applied the analytical framework to each study reviewed by writing the corresponding number directly onto the findings (i.e., themes, subthemes, direct quotes) of each study. This step then involved charting (i.e., moving the findings from its original textual context and placing it in the framework). Sixth, themes and subthemes were interpreted and a thick description of each theme was developed with supporting quotations selected from the original studies to build a complex, holistic picture [31]. This was then reviewed by a fifth author (Jennifer Brunet) and discussed amongst all authors.

The second and third main analytical steps involved an analysis of the methodological (i.e., meta-method analysis) and conceptual/theoretical approaches (i.e., meta-theory analysis) used to explore their appropriateness, and importantly their influence on the findings [25]. Analysis of the meta-method focused on the design features of the study, sampling techniques, data collection procedures, and analytical techniques. Analysis of the meta-theory focused on the research paradigms, theoretical assumptions, and conceptual/theoretical underpinnings.

## 3. Results

### 3.1. Search Results

**Figure 1:**
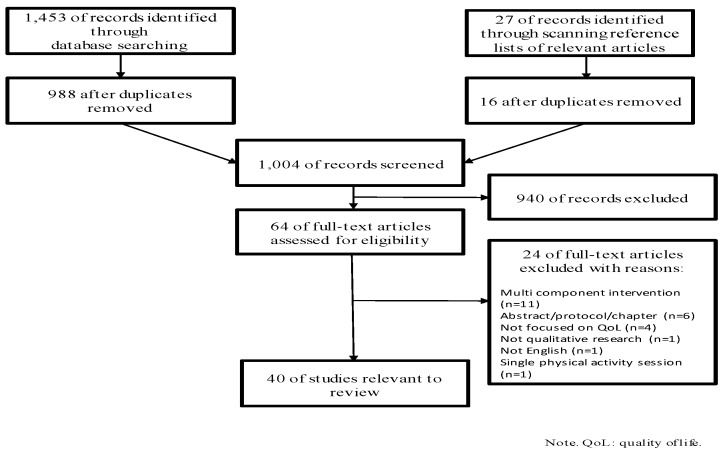
Flow diagram of studies identified, screened, and included in this meta-synthesis, along with reasons for exclusion.

The electronic database and hand-searches yielded 1480 citations. After removing duplicates, 1004 citations remained for title and abstract screening. Sixty-four studies were identified as potentially relevant and were subsequently assessed for eligibility through full-text screening. Of these, 40 studies reporting qualitative findings related to the impact of physical activity on QoL in cancer survivors met eligibility criteria and were included in this review. Figure 1 presents a flow chart of the numbers of retrieved, included, and excluded studies at different phases of the screening process, along with reasons for exclusion.

### 3.2. Study Characteristics

Table 1 provides a summary of the characteristics for the 40 studies included in this review. They were published between 2004 and 2016. There were a total of 604 participants included, with the average sample size being 15 participants. There was a wide range in participants’ age (i.e., 32 to 90 years; *n* = 11 not reported), and the mean age across studies was 57.3 years (*n* = 12 not reported). The majority of participants were women (81%), and just under half of the studies included women diagnosed with breast cancer (*n* = 19). The remaining studies included men and/or women who had been diagnosed with colorectal (*n* = 2), gynaecological (*n* = 2), lung (*n* = 1), multiple myeloma (*n* = 1), or prostate cancer (*n* = 4). Other studies were not specific to a specific type of cancer (i.e., mixed cancers; *n* = 11). Most were conducted with adults who were off-treatment (*n* = 17), and an equal number of studies were conducted with adults who were on-treatment (*n* = 9) or with adults either on-treatment or off-treatment (*n* = 9); the remaining studies did not specify treatment status (*n* = 5).

Participants engaged in physical activity interventions lasting 13.9 weeks on average (*n* = 14 not reported) and there was some variability in terms of the type of physical activity across studies. The types included: dragon boating (*n* = 7), high altitude trekking (*n* = 2), Nordic walking (*n* = 1), recreational football (*n* = 1), structured resistance training and/or aerobic activity (*n* = 14), unstructured walking (*n* = 1), and yoga/mindful movement (*n* = 8); type(s) was/were not reported in 6 studies. Most consisted of group-based activities (*n* = 25); the remaining consisted of individual-based activities (*n* = 9) or a combination of group- and individual-based activities (*n* = 6).

A variety of methods were used to collect qualitative data. The majority of studies used individual interviews (*n* = 21). The rest used focus groups (*n* = 10), multiple methods (e.g., interviews and observations; *n* = 8), or mailed open-ended questionnaires (*n* = 1). Content analysis (*n* = 11) and phenomenological analysis (*n* = 10) were used to analyze the data in most studies. Other analytical techniques used included: case study analysis (*n* = 1), categorical aggregation (*n* = 1), constant comparison (*n* = 1), framework analysis (*n* = 5), grounded theory (*n* = 1), mixed data analysis techniques (*n* = 1), systematic text condensation (*n* = 1), and thematic analysis (*n* = 6). Two studies did not report which method(s) was/were used to analyze the data.

In general, details provided regarding the study aims, sample characteristics, and method(s) used to collect data were sufficient to allow readers to be able to replicate the study. However, details on the underpinning theoretical/conceptual frameworks (*n* = 28 not reported) and ontological/epistemological approaches (*n* = 33 not reported) used to guide study design and data analysis were lacking in most studies. Of the studies that did provide details on theoretical/conceptual frameworks used, body image (*n* = 1), cancer-related fatigue (*n* = 1), mindfulness (*n* = 1), QoL (*n* = 2), posttraumatic growth (*n* = 2), social cognitive theory (*n* = 1), social support (*n* = 2), theory of explanatory models (*n* = 1), and well-being/wellness (*n* = 4) were cited as the guiding theoretical/conceptual frameworks. Ontological/epistemological approaches that were cited included: constructivism (*n* = 3), feminism (*n* = 1), and non-realism (*n* = 3).

### 3.3. Main Results

Table 2 presents the themes and subthemes that resulted from the meta-data analysis, along with supporting quotations from the original studies. Overall, the analysis of the data showed that cancer survivors’ viewed physical activity as having a positive impact on their QoL. Due to the interconnected and dynamic nature of the multiple dimensions of QoL, the themes and subthemes identified were not exclusive, but rather were overlapping and mutually reinforcing.

#### 3.3.1. Physical Well-Being

Across studies, participants reflected on specific physical benefits they experienced through physical activity. They mainly noticed improvements in their physical functioning and health. They also felt that physical activity helped them to manage the physical consequences of cancer and its treatment, which contributed to their overall physical QoL.

*Improved physical and functional health*. Physical activity helped participants feel physically better, ward off perceived health concerns, and function better in their day-to-day life. Participants noted specific benefits in terms of their physical fitness [38,40,41,44,46,48,50,52,53,54,58,59,64,65,69], overall energy levels [32,33,35,37,38,40,42,43,44,46,48,50,54,56,57,58,59,62,65,66,67], physical strength [32,35,42,43,44,46,49,50,51,54,56,57,59,60,61,62,66,68,69], flexibility [38,39,49,56,67], weight/body composition [42,43,53,54,62,69,70], sleep quality [40,43,56,61,66], functional mobility [32,35,36,37,46,54,58,60,62,64,65], pain/discomfort [36,37,38,45,56,62], ability to relax [49,56,57,61], ability to engage in tasks requiring coordination [32,67], and overall physical well-being [69]. Yet, some participants had negative experiences [32,37,54,70]. For example, Backman et al. [32] noted that women who were receiving adjuvant chemotherapy treatment for breast cancer experienced nausea, lethargy, and headaches when participating in aerobic and/or resistance training. Additionally, others experienced feelings of fatigue, though some noted that feeling tired was a good thing because they viewed as a “healthy fatigue” [54,68].

*Managing the physical consequences of cancer and its treatment*. Participants described how physical activity helped them alleviate the adverse physical effects they attributed to the disease and its treatment. In particular, physical activity helped participants manage their cancer-related fatigue [41,52,61,67] and pain [33,36,38,40,61,66], as well as improve their overall physical appearance [33,43].

Some participants also explained that physical activity made them feel like they were self-managing their disease by reducing the risk of their cancer reoccurring [33,45,49,60,69], decreasing their risk of developing a new cancer [33,45,49,60,69], or by slowing down the progression of their disease and prolonging their life. Other participants spoke more generally about how physical activity relieved various adverse consequences of their treatments [33,40,56,57].

#### 3.3.2. Psychological Well-Being

The theme covering the psychological benefits of physical activity for cancer survivors was the most diverse theme identified. It was comprised of five specific aspects that contributed to overall psychological/emotional QoL. These included focusing on health rather than illness, (re)discovering strength and physical capabilities, exercising control and taking action, evoking positive self-perceptions and minimizing negativity, and gaining a sense of normalcy.

*Focusing on health rather than illness*. Physical activity enabled participants to shift their focus from sickness and disease to wellness and health [32,33,38,39,42,45,48,50,53,54,55,57,61,63,67,69]. It also gave them something to do and kept their minds busy. For example, amongst many participants undergoing treatment, physical activity provided relief from their preoccupations with their illness and served as a break from being consumed by their disease e.g., [40,41]. For participants who had completed treatment, physical activity helped them gain closure by creating a distance from their previous experiences with cancer thereby supporting the transition from being ill to being well e.g., [35].

*(Re)discovering strength and physical capabilities*. Physical activity provided opportunities for participants to (re)discover what their bodies were capable of doing, regardless of where they were along the cancer trajectory (e.g., on-treatment, off-treatment [34,41,42,43,45,46,47,48,49,50,51,52,53,55,59,62,66,67,68,69,71]. It also fostered body awareness by helping some participants (re)gain a connection with their bodies. Moreover, many focused on the benefits of challenging themselves to engage in physically demanding tasks. For those who had experienced physical health declines since their diagnosis, it made them realize that they were physically strong and capable. These experiences helped participants to (re)gain trust in their bodies and allowed them to (re)define themselves as physically strong and able persons.

*Exercising control and taking action*. Physical activity was viewed by participants as something they could do to promote their own health, which fostered a sense of control over at least one facet of their lives [33,35,40,41,45,47,48,50,51,52,55,62,63,65,68,69]. Because many participants commented that they felt as though they had lost control over their health and body since their diagnosis, doing activities that made them feel in control and empowered was important. Physical activity served this purpose and enhanced their desire to assume even more responsibility for improving their lives.

*Evoking positive self-perceptions and minimizing negativity*. Physical activity fostered a range of positive self-perceptions. Participants used specific terms such as ‘proud’, ‘accomplished’, and ‘confident’ to describe how they felt about themselves after physical activity [32,33,34,35,36,38,39,40,44,46,52,54,55,56,57,58,59,60,62,63,65,66,67,69,70]. They also expressed more general positive feelings. For example, Cormie et al. [69] reported that men with prostate cancer felt ‘better’ about themselves. Similarly, Coon and Coleman [67] reported that adults with multiple myeloma felt ‘better’ about themselves after physical activity. In addition, physical activity helped participants ward off negative feelings and thoughts. Bulmer et al. [33] reported that women with breast cancer felt less anxious and depressed after aerobic and resistance training, and were able to better manage stressful aspects of cancer such as fear of dying. Further, Van Puymbroeck et al. [49] reported that yoga helped women with breast cancer manage various cancer-specific and general stressors (e.g., work-related stresses).

*Gaining a sense of normalcy*. A prevailing notion across studies was that participants wanted to feel “normal” again and that physical activity helped them acquire this feeling. Parry [45] provided evidence of this and reported that as women with breast cancer engaged in dragon boating, they felt normal at a time when they were adjusting to new and unfamiliar emotional and physical changes after treatment. This finding was supported by Backman et al. [32] who found that physical activity during adjuvant chemotherapy treatment helped women with breast cancer feel like life continued in a normal way. Overall, physical activity provided participants with opportunities to get back to what they were doing before their diagnosis and/or offered them an opportunity to engage in activities of ‘normal’ life [32,33,39,40,41,45,50,61,62,64,67].

#### 3.3.3. Social Well-Being

Participants experienced social benefits by participating in physical activity. Improvements in social interactions and networks, feeling understood by others, having stronger social connections with others, and being able to give and receive support were mainly reported. These specific benefits contributed to cancer survivors’ overall social QoL.

*Feeling understood by others*. Because many participated in group-based activities with other cancer survivors, participants had the opportunity to be around other survivors, and as a result, many felt understood in a social context characterized by reciprocal approval and recognition that was different from traditional support groups [33,35,37,41,42,43,44,45,46,47,48,49,50,55,60,61,62,63,64,65,69]. Participants were able to socialize without having to explicitly talk about cancer and appreciated the unspoken shared connection amongst them. For example, Luoma et al. [41] reported that women with breast cancer felt understood by other group members even though they did not talk about cancer while participating in group-based physical activity. For many, it was important that physical activity was the focus, not cancer. However, it is important to note that some participants had concerns about participating in physical activity with other cancer survivors [37,41,42,48], either because they had to deal with the death of a group member [47] or because it constantly reminded them that they had had cancer [42].

*Fostering social connections*. For many participants, the physical activity context facilitated social connections between themselves and others [34,37,40,41,42,43,44,45,46,47,50,52,53,54,55,59,61,62,63,64,65,68,69], mostly because it provided an opportunity for them to make new connections. Further, physical activity helped to reduce feelings of social isolation and increased feelings of relatedness, belongingness, and camaraderie. Cormie et al. [69] found that men diagnosed with prostate cancer made connections with other people at the gym and felt cared for. Extending beyond the physical activity context, participants believed their participation in physical activity enhanced their interactions with family members and friends e.g., [51]. Nevertheless, negative social interactions were conveyed by some women with breast cancer who had experienced a few aversive, problematic social conflicts with women while dragon boating, which detracted from their social well-being and hindered the social camaraderie they experienced [42].

*Giving and receiving support*. Physical activity provided opportunities for participants to give and receive support [33,35,36,37,41,42,43,45,46,47,48,50,54,57,60,61,62,64,65,68,69]. The physical activity contexts were characterized by mutual encouragement, which offered participants opportunities for informal counseling/advice and support from other cancer survivors. For example, women with breast cancer received informational support through informal conversations from other women after the physical activity classes e.g., [41,42]. Some participants also reported gaining support from other individuals who were part of the group but who were not always diagnosed with cancer such as the instructor or support persons.

#### 3.3.4. Spiritual Well-Being

Physical activity was seen as facilitating a spiritual awakening among participants. Specific spiritual benefits included (re)defining life purpose and living meaningfully and becoming mindful, which contributed to overall spiritual QoL.

*(Re)defining life purpose and living meaningfully*. Physical activity fostered a sense of direction, purpose and coherence in the day-to-day lives of participants [33,35,36,42,45,46,47,51,55,56,60,62,65,69,71]. For example, Burke et al. [51] found that pre-surgical exercise training provided adults with advanced rectal cancer direction and purpose as they awaited surgery. Sabiston et al. [47] reported that dragon boating helped women with breast cancer find life meaning by filling a void that cancer had created. Physical activity also helped participants achieve a greater sense of life meaning by helping participants feel like their life had value.

*Becoming mindful*. Physical activity encouraged participants to connect with their minds and bodies on a deeper level [34,36,45,46,48,55,56,57,61,66,68,69]. Their involvement in physical activity represented an opportunity to live in the moment. For example, Crane-Okada et al. [36] found that mindful movement exercise enabled older women with breast cancer to slow down and experience a heightened awareness of their bodies. Moreover, Ray and Verhoef [46] found that dragon boating helped women with breast cancer stay focused on the present moment.

## 4. Discussion

The aim of this review was to collate existing qualitative research examining cancer survivors’ perspectives regarding the impact of physical activity on their QoL using an efficient and rigorous scientific approach (i.e., meta-synthesis). The 40 studies that were reviewed, which included men and women diagnosed with various types of cancers, provide convincing evidence that physical activity yields a range of perceived benefits that can be categorized under four broad dimensions of QoL: physical, psychological/emotional, social, and spiritual. Synthesizing cancer survivors’ personal accounts of their experiences of QoL led to the generation of rich data and in-depth descriptions about the impact of physical activity in a way that other types of systematic reviews (e.g., quantitative meta-analyses) have been unable to reveal. Importantly, this review suggests that physical activity is a promising strategy for helping cancer survivors manage the adverse side effects of cancer and its treatments, focus on their health rather than their illness, rediscover strength and physical abilities, feel normal, foster social connections and support, live meaningfully, and become mindful. Further, it corroborates current conceptualizations of QoL [72,73] by showing that QoL is not a static, unidimensional construct; rather, it is a subjective, broad, and multidimensional construct that includes different dimensions that are dynamic such that changes in one dimension (e.g., physical well-being) can influence other dimensions (e.g., psychological/emotional well-being) [74].

This review helped to uncover areas that need to be explored in future research. Although generally positive, there appears to be differences in cancer survivors’ views of the impact of physical activity on specific aspects of their QoL depending on various characteristics. Specifically, cancer survivors’ accounts of the impact of physical activity on them and their lives seemed to vary depending on where they were at along the cancer continuum (e.g., on- vs. off-treatment) and depending on the stage of their disease (e.g., early- vs. advanced-stage). On the one hand, physical activity helped cancer survivors’ feel healthy and strong once they had finished treatment e.g., [37,45]. On the other hand, physical activity provided a distraction from the disease and structure in their day amongst those who were undergoing active treatment e.g., [60]. Additionally, physical activity helped cancer survivors who were diagnosed with early stage cancer restore their sense of wellness and improve daily functioning e.g., [40], whereas it helped those diagnosed with progressive, advanced stage cancer feel like they were slowing the progression of their disease and prolonging their life e.g., [56,63]. Identifying these potential sources of heterogeneity across studies in terms of stage of disease and treatment highlights the importance of including adults with early and advanced-stage cancer who are at different phases along the cancer trajectory and considering the influence of these factors in future research.

This review also highlights the need to explore whether cancer survivors view different types of physical activity as having a different impact on their QoL. Physical activity is recognized as a complex behaviour that includes leisure-time, occupational, commuting, and household activities [75], and there is growing recognition of the importance of distinguishing between these activities. However, this review also suggests there may be differences in cancer survivors’ experiences of QoL depending on the type of leisure-time physical activity. On the one hand, leisure-time physical activity that emphasized mind-body connections such as yoga seemed to instill an improved ability to relax and experience mindfulness e.g., [56,61]. On the other hand, adventurous physical activity that was strenuous, aerobic, and/or strength-based such as dragon boating, scaling Mt. Kilimanjaro, and interval cycling seemed to improve perceptions of strength and physical fitness e.g., [35,43,44,51]. Accordingly, further research is needed to explore if different types of leisure-time physical activity have a different meaning and utility for cancer survivors’ and how this might impact their experiences of QoL.

Another notable area for future research includes examining cancer survivors’ views on how the dosage (i.e., frequency, intensity, duration) and setting in which physical activity is delivered (e.g., home, hospital, community centre) may impact their QoL. Few studies reviewed herein or in previous quantitative reviews [11,12,13,14,15,16,17,18] have focused on establishing which specific contexts or parameters of physical activity may be most effective in positiveily impacting survivors’ experiences of their QoL. Although there is some evidence from qualitative research that breast and advanced rectal cancer survivors felt that having physical activity supervised and offered in hospitals made them feel safe and secure while exercising [32,51], failure to further evaluate survivors’ experiences across different contexts and/or parameters of physical activity currently prevent specific recommendations for programming. Similarly, though offering group-based physical activity may be especially beneficial, based on the qualitative evidence, as it provided cancer survivors opportunities to interact with and receive support from others e.g., [62,68], comparisons within studies of individual- and group-based physical activity are lacking. Thus, in line with Brown et al.’s [76] conclusion, cancer survivors’ perceptions of the specific dosage of physical activity needed to optimally improve QoL and facilitate symptom management needs to be investigated further.

Although this review helps to establish that both men and women viewed physical activity as having a positive impact on various aspects of their QoL post-diagnosis, the lack of studies focused on men remains a noteworthy gap in the literature. Considering that women consisted of 81% of the sample across the 40 studies reviewed, more initiatives to recruit men are needed. This is especially important because men also experience significant personal and external barriers to participating in cancer support groups [73,77]. Moreover, the limited body of quantitative research on sex differences and QoL outcomes (e.g., pain, fatigue, and depression) has yielded conflicting results [77].

### 4.1. Practical Implications

Promoting cancer survivors’ subjective evaluation of their well-being and functioning across multiple QoL domains is a priority because it is associated with clinical outcomes [9]. Physical activity is increasingly being used as a strategy to enhance cancer survivors’ experiences of QoL. This review of 40 published qualitative studies in this area showed that physical activity can enhance physical, psychological/emotional, social and spiritual dimensions of QoL. This collective body of research, coupled with the evidence from previous quantitative reviews e.g., [13,14,15,16,17], provides a strong scientific basis for its recommendation as an adjunct to cancer care to improve multiple dimensions of QoL. Given recent emphasis on enhancing self-management among cancer survivors [78], promoting physical activity in clinical practice may be one way to encourage cancer survivors to take more responsibility for their health.

Healthcare practitioners are uniquely suited to do this by prescribing physical activity [18]. To increase the likelihood that cancer survivors will follow healthcare practitioners’ recommendation/prescription, physical activity that is appropriate for the patient depending on their individual needs should be prescribed. That is, certain types of physical activity may be better suited depending on the specific QoL issues present in the lives of their patients’. For example, to mitigate stress and promote relaxation, healthcare practitioners may want to recommend yoga. When recommending physical activity, healthcare practitioners can make use of existing assessments which ensure cancer survivors are physically able to participate (i.e., PAR-Q+ ePARmed-X+ [79]). Likewise, healthcare practitioners need to be cognizant of possible negative experiences with physical activity participation. Indeed, this review helped discover that a small number of cancer survivors may experience adverse effects including shortness of breath, dizziness, weight gain, and interpersonal conflict.

### 4.2. Implications for Future Research

In the past several decades, there has been an increased focus on exploring cancer survivors’ perspectives on the impact of physical activity on QoL in studies using interviews, focus groups, and observations. Though much has been learned, various gaps are apparent based on the current review that should be addressed in future research using qualitative methods. First, the majority of studies focused on middle-aged women diagnosed with breast cancer who had completed treatment. Researchers need to closely examine other groups of adult survivors (e.g., men, young adults, elderly) and those at different points along the cancer trajectory (i.e., on-treatment, palliation) as this may reveal different QoL experiences related to physical activity. Second, further work is needed to develop theoretical models related to physical activity and QoL for cancer survivors. Similarly, more attention needs to be focused on exploring QoL comprehensively as a multidimensional concept that includes subjective evaluations of well-being across multiple life domains in order to better understand the processes underlying survivors’ experiences of their QoL. In this way, researchers may want to consider using more diverse qualitative methodologies (e.g., ethnography, narrative discourse, grounded theory). Last, as qualitative research is becoming increasingly accepted and published in various journals, the necessity for high quality qualitative studies (or mixed/multi-method studies in which qualitative data are collected) should not be underestimated. In this respect, published guidelines for developing, implementing, and disseminating findings from qualitative studies should be followed to ensure important issues are not overlooked and that studies produce meaningful and trustworthy findings [26,27].

Encouraging findings were reported in the studies reviewed herein; yet, as mentioned above, it is unclear how dosage (i.e., intensity, frequency, and duration) and type of physical activity impacts on cancer survivors’ experiences of QoL. It is also unclear if the dosage and type of physical activity should be prescribed to promote physical, psychological/emotional, social, and spiritual functioning or if these should be self-selected by cancer survivors. In most studies, participants were instructed to adhere to a specific intervention (e.g., moderate intensity physical activity such as cycling for 30 minutes, 3 times per week, during an 8-week period). However, an emerging approach in the general population is to allow participants to self-select the intensity, frequency, and duration of physical activity, and that this can lead to better psychological/emotional functioning [80]. Thus, future studies are needed to confirm if this would also be the case in cancer survivors. As well, studies with longer follow-up assessments are warranted to determine if improvements in QoL are sustained over time.

### 4.3. Strengths and Limitations

A main strength of this meta-synthesis is that a comprehensive approach following recommendations for meta-analyses as outlined by Paterson et al. [25] was taken to analyze, synthesize, and interpret qualitative findings presenting in studies exploring cancer survivors’ perspective of the impact of physical activity on their QoL. Having multiple authors independently screen, extract, analyze, and interpret findings from retrieved studies and developing the search strategy in consultation with an experienced librarian are also strengths. Nevertheless, there are notable limitations that should be considered. First, only peer-reviewed published studies were reviewed. Thus, the risk of publication bias, whereby studies showing a beneficial impact of physical activity may have been more likely to be published, should be taken into account. Second, only studies published in English language were reviewed. Third, though the data analysis was conducted by multiple authors, the themes and subthemes developed herein may be different from those developed by other authors.

## 5. Conclusions

This review used rigorous methods (i.e., meta-synthesis) to synthesize a large body of qualitative research and showed that cancer survivors’ view physical activity as positively impacting various dimensions of their QoL. It represents a much-needed synthesis of this research as prior reviews have focused on quantitative evidence e.g., [13,14,15,16,17]. From the 40 studies reviewed, four main themes characterized by a focus on survivors’ experiences of their physical, psychological/emotional, social, and spiritual well-being were identified and ultimately help to extend our understanding of how physical activity impacts the lives of cancer survivors. Based on this review, physical activity may promote experiences of QoL by helping cancer survivors feel more satisfied physically and psychologically/emotionally, more socially connected and supported, and live meaningfully and mindfully. Interventions promoting physical activity are likely to have significant implications for promoting positive changes in cancer survivors’ QoL, and should therefore feature in future studies and practice seeking to find alternative therapies to promote QoL in this population.

## Figures and Tables

**Table 1:** Study characteristics.

Study	Country	Aim(s)	Sample	Intervention	Data Collection Method(s)	Design	Method/Analysis	Theoretical and/or Conceptual Orientation	Quality Rating
**Breast Cancer (*n* = 19 studies)**									
Backman et al. [32]	SWE	Explore women’s experiences of physical activity during adjuvant chemotherapy treatment	16 women Treatment status: On-treatment Age range: 36–70 years Mean age: 54.0 years (individual interviews), 64.0 years (focus group)	16-weeks of structured, individualized, aerobic and/or a mix of resistance and aerobic training	Interviews and focus group	Observational (cross-sectional) [part of a larger experimental (randomized controlled trial)]	Qualitative/Inductive content analysis	Not stated	High
Bulmer et al. [33]	USA	Describe women’s perceptions of the benefits of participation in an individualized exercise service	45 women Treatment status: On- and off-treatment Age range: 32–64 years Mean age: 53.1 years	Structured and unstructured, individualized, aerobic and resistance training	Interviews and email journals	Observational (cohort)	Qualitative/Thematic analysis	Not stated	Medium
Burke & Sabiston [34]	CAN	Explore women’s lived experiences scaling Mt. Kilimanjaro	3 women Treatment status: Off-treatment Age range: 43–63 years Mean age: not stated	9-days of high altitude trekking	Interviews and observations	Observational (cohort)	Qualitative (ethnography)/Case study analysis	Post- traumatic growth, Non realist paradigm	High
Burke & Sabiston [35]	CAN	Explore experiences of subjective well-being among women attempting to scale Mt. Kilimanjaro	6 women Treatment status: Off-treatment Age range: 43–63 years Mean age: 52.5 years	9-days of high altitude trekking	Interviews and observations	Observational (cohort)	Qualitative (phenomenology)/Hermeneutic phenomenological analysis	Subjective well-being, Non realist paradigm	High
Crane-Okada et al. [36]	USA	Examine women’s perceptions of the effects of a mindful movement program on QoL and mindfulness	16 women Treatment status: Off-treatment Age range: 51–90 years Mean age: 66.3 years	12-weeks of mindful movement exercises	Focus groups	Observational (cross-sectional) [part of a larger experimental (randomized controlled trial)]	Qualitative/Content analysis	Mindfulness and movement	Medium
Fischer et al. [37]	NLD	Investigate the impact of a Nordic walking intervention on women’s subjective well-being and shoulder functioning	28 womenTreatment status: Off-treatment Age range: 36–75 years Mean age: 53.8 years	10-weeks of structured Nordic walking	Focus groups	Quasi-experimental (case series)	Mixed methods/Inductive content analysis	Not stated	Low
Galantino et al. [38]	USA	Evaluate the impact of yoga on functional outcomes, pain, and health-related QoL for postmenopausal women with aromatase inhibitor-associated arthralgia	10 women Treatment status: On-treatment (i.e., aromatase Inhibitors) Age range: 50–71 years Mean age: 58.0 years	8-weeks of structured community-based yoga classes and home-based practice	Journal entries and phone calls	Quasi-experimental (case series)	Qualitative/Content analysis	Social cognitive theory	Medium
Galantino et al. [39]	USA	Identify the impact of yoga on cognition, functional outcomes, and QoL	4 women Treatment status: On-treatment Age range: 44–65 years Mean age: 54.8 years	12-weeks of structured yoga classes and home-based practice	Mailed open-ended questions	Quasi-experimental (case series)	Mixed methods/Content analysis	Not stated	Low
Husebø et al. [40]	NOR	Describe women’s perceptions of a home-based exercise intervention during chemotherapy on physical and psychosocial wellness	27 women Treatment status: On-treatment Age range: 34–69 years Mean age: 52.0 years	19-weeks of structured, home-based aerobic (i.e., walking) and resistance training	Focus groups	Quasi-experimental (case series)	Qualitative/Systematic text condensation	Wellness	Medium
Luoma et al. [41]	FIN	Investigate women’s experiences of participating in a tailored exercise intervention	25 women Treatment status: Off-treatment Age range: 43–67 years Mean age: 54.0 years	52-weeks of structured group-based aerobic training and home-based aerobic training	Focus groups	Observational (cross-sectional) [part of a larger experimental (randomized controlled trial)]	Qualitative/Phenomenological analysis	Not stated	Medium
McDonough et al. [42]	USA	Explore women’s experiences of changes in their body image and feelings of social support during a novice season of dragon boating	14 women Treatment status: Off-treatment Age range: 46–60 years Mean age: 54.2 years	~12-weeks (i.e., a single season) of dragon boating	Interviews	Observational (cohort)	Qualitative (phenomenology)/Interpretative phenomenological analysis	Body image, Social support	High
McDonough et al. [43]	USA	Explore the development of social relationships, social support, and outcomes among women participating in a dragon boating program over two seasons	17 women Treatment status: Off-treatment Age range: not stated Mean age: 51.2 years	~76-weeks (i.e., two season) of dragon boating	Interviews	Observational (cohort)	Qualitative/Interpretative phenomenological analysis	Social support, Post- traumatic growth	High
Mitchell et al. [44]	CAN	Explore the expectations, experiences, and psychosocial impact of dragon boating from the perspective of new members	10 women Treatment status: Off-treatment Age range: 35–70 years Mean age: not stated	1 season of dragon boating	Interviews	Observational (cohort)	Qualitative/Thematic analysis	Constructivist paradigm, Community-based participatory research approach	High
Parry [45]	CAN	Understand how participation in dragon boating contributes to women’s health throughout survivorship	11 women Treatment status: Off-treatment Age range: Mid 40’s–early 60’s Mean age: not stated	Dragon boating	Interviews	Observational (cohort)	Qualitative/Constant comparison	Feminist epistemeology, holistic philosophical perspective	High
Ray and Verhoef [46]	CAN	Explore women’s lived experience of dragon boating and how and why this experience is perceived to influence their health-related QoL	15 women Treatment status: Off-treatment Age range: not stated Mean age: not stated	1 season of dragon boating	Interviews	Observational (cross-sectional)	Mixed methods/Content analysis	Health-related QoL	High
Sabiston et al. [47]	CAN	Explore women’s experiences of engaging in dragon boating	20 women Treatment status: Off-treatment Age range: 42–70 years Mean age: 58.7 years	Dragon boating	Interviews	Observational (cross-sectional)	Qualitative/Grounded theory	Constructivist paradigm, Post-positivist approach	High
Unruh & Elvin [48]	CAN	Explore the impact of dragon boat racing on psychological well-being	3 women Treatment status: Off-treatment Age range: Early 50’s Mean age: not stated	Dragon boating	Interviews and field notes	Observational (cohort)	Qualitative/Content and thematic analysis	Psychological well-being	High
Van Puymbroeck et al. [49]	USA	Describe the health benefits of participation in a yoga intervention	18 women Treatment status: Off-treatment Age range: not stated Mean age: not stated	8-weeks of structured, community-based yoga sessions and home-based practice	Focus groups	Observational (cross-sectional) [part of a larger experimental (randomized controlled trial)]	Qualitative (phenomenology)/Interpretive phenomenological analysis	Not stated	Medium
Wurz et al. [50]	CAN	Explore the barriers and motives experienced by women attending a physical activity program offered in the community	7 women Treatment status: Off-treatment Age range: not stated Mean age: 55.3 years	8-weeks of structured, group-based training	Interviews	Observational (cohort)	Qualitative/Thematic analysis	Not stated	High
**Colorectal Cancer (*n* = 2)**									
Burke et al. [51]	GBR	Explore participants’ perceptions of QoL during a structured, hospital-based preoperative exercise program	7 women, 3 men Treatment status: On-treatment Age range: 45–74 years Mean age: 58.2 years	6-weeks of structured, aerobic (i.e., cycling) interval training	Interviews	Quasi-experimental (case control)	Qualitative (phenomenology) Hermeneutic phenomenological analysis	QoL, Non-realist paradigm	High
Spence et al. [52]	AUS	Document participants’ experiences in an exercise rehabilitation program and their preferences for program content and delivery	3 women, 7 men Treatment status: Off-treatment Age range: 42–74 years Mean age: not stated	12-weeks of supervised, individualized aerobic training	Interviews	Quasi-experimental (case Qualitative/ Thematic series) analysis	Not stated	Medium	
**Gynecologic Cancer (*n* = 2)**									
Donnelly et al. [53]	GBR	To determine the feasibility and efficacy of a physical activity behavioral change intervention in managing cancer-related fatigue	33 women (*n* = 16; focus group) Treatment status: On- and off-treatment Cancer diagnosis: Endometrial, ovarian Age range: not stated Mean age: 53.0 years	12-weeks of structured, home-based aerobic (i.e., walking) and resistance training	Focus groups	Observational (cross-sectional) [part of a larger experimental (randomized controlled trial)]	Mixed methods/Framework analysis	Not stated	Low
Donnelly et al. [54]	GBR	Explore perceptions and experiences of participation in a randomized controlled trial (Donelley et al., [53]) testing the efficacy of a home-based physical activity intervention	16 women Treatment status: On- and off-treatment Cancer diagnosis: Endometrial, ovarian Age range: 38–78 years Mean age: 55.0 years	12-weeks of home-based aerobic (i.e., walking) and resistance training	Focus groups	Observational (cross-sectional) [part of a larger experimental (randomized controlled trial)]	Qualitative/Framework analysis	Not stated	Medium
**Lung Cancer (*n* = 1)**									
Missel et al. [55]	DNK	Explore the perceived benefits and barriers to participating in a postoperative, community-based exercise intervention	11 women, 8 men Treatment status: On- and off-treatment Age range: 48–75 years Mean age: 63.0 years	12-weeks of structured aerobic and resistance group-based training	Interviews	Observational (cohort) [part of a larger experimental (randomized controlled trial)]	Qualitative/Hermeneutic phenomenological analysis	Not stated	Medium
**Mixed Cancer (*n* = 11)**									
Carr et al. [56]	CAN	Develop an understanding of the potential physical and psychosocial impact of yoga on the well-being for participants with end stage cancer	3 women Treatment status: Not stated Cancer diagnosis: Breast, lymphatic system Age range: 60’s Mean age: not stated	3 home-based yoga sessions	Interviews	Quasi-experimental (case series)	Qualitative/Content analysis	Well-being	Medium
Duncan et al. [57]	CAN	Explore the benefits of Iyengar	23 women, 1 man (*n* = 6; interviews) Treatment status: On- and off-treatment Cancer diagnosis: Breast, gynecologic; lymphatic system Age range: not stated Mean age: 49.3 years	10-weeks of structured yoga sessions	Interviews	Quasi-experimental (case series)	Mixed-methods/Categorical aggregation	Not stated	Low
Frensham et al. [58]	AUS	Explore experiences of rural participants engaging in an online lifestyle intervention	6 women, 2 men Treatment status: Off-treatment Cancer diagnosis: breast, bowel, lymphatic system, prostate Age range: 43–78 years Mean age: 67.0 years	6-weeks of online pedometer-based walking	Interviews	Quasi-experimental (case series)	Qualitative/Content analysis	Not stated	Medium
Groeneveld et al. [59]	NLD	Explore participants’ experiences with returning to work and participating in an exercise program	9 women, 1 man Treatment status: Off-treatment Cancer diagnosis: breast, bone marrow, ovary Age range: 39–60 years Mean age: 56.0 years	12-weeks of structured aerobic (i.e., cycling) and resistance group-based training	Interviews	Quasi-experimental (case series)	Qualitative (phenomenology)/Not stated	Not stated	Low
Gulde et al. [60]	SWE	Explore experiences of physical activity among participants with end stage cancer	6 women, 5 men Treatment status: Not stated Cancer diagnosis: breast, cervical, colon, brain, ovary, pancreas, prostate, renal, ventricular Age range: 45–81 years Mean age: 61.4 years	Structured physical activity sessions	Interviews	Observational (cross sectional)	Qualitative/Content analysis	Not stated	Medium
Mackenzie et al. [61]	CAN	Explore the benefits of a community-based yoga program among survivors and their support persons	22 women, 3 men (20 survivors; 5 support persons) Treatment status: Not stated Cancer diagnosis: breast, cervical, colorectal, lymphatic system, ovary, prostate Age range: not stated Mean age: 56.1 years (survivors); 71.8 years (support persons)	7-weeks of community-based yoga sessions	Focus groups	Quasi-experimental (case series)	Qualitative (phenomenology)/Inductive thematic analysis	Not stated	Medium
McGrath et al. [62]	AUS	Examine the psychosocial benefits of participating in an exercise club designed for chemotherapy patients	6 women, 3 men Treatment status: On- and off-treatment Cancer diagnosis: lymphatic system, breast, colorectal, ovary Age range: 57–74 years Mean age: not stated	Hospital-based, supervised, individualized, training	Interviews	Observational (cross-sectional)	Qualitative/Not stated	Not stated	Medium
Paltiel et al. [63]	NOR	Explore the meaning of group-based exercise for participants with end stage cancer	2 women, 3 men Treatment status: On-treatment (*n* = 3) Cancer diagnosis: colon, ovary, soft tissue sarcoma, rectum Age range: 42–76 years Mean age: not stated	6-weeks of structured, group training	Interviews	Quasi-experimental (case series)	Qualitative (phenomenology)/Hermeneutic phenomenological analysis	Not stated	High
Stevinson and Fox [64]	GBR	Evaluate the feasibility and acceptability of a group-based exercise program	7 women, 5 men Treatment status: On- and off-treatment Cancer diagnosis: breast, lung, lymphatic system, ovary, prostate Age range: 43–73 years Mean age: 59.0 years	10 weeks of structured aerobic and resistance circuit training supplemented by home-based activity	Interviews and participant diaries	Quasi-experimental (case series)	Qualitative/Framework analysis	Not stated	Medium
Turner et al. [65]	GBR	Investigate participants’ experiences of hospice-based exercise	7 women, 2 men Treatment status: Not stated Cancer diagnosis: brain, breast, lung, lymphatic system, bone marrow, ovary, pancreas, prostate Age range: 55–82 years Mean age: not stated	Hospice-based, individualized, aerobic and resistance training	Interviews	Observational (cross-sectional)	Qualitative (phenomenology)/Interpretive phenomenological analysis	Not stated	High
van Uden-Kraan et al. [66]	DNK	Explore participants’ motives for and experiences of practicing yoga	25 women, 4 men Treatment status: Not stated Cancer diagnosis: brain breast, colorectal, endometrial, lymphatic system, kidney, lung, Age range: not stated Mean age: 53.8 years	Structured yoga classes	Focus groups	Observational (cross-sectional)	Qualitative/Thematic framework analysis	Not stated	Medium
**Multiple Myeloma (*n* = 1)**									
Coon & Coleman [67]	USA	Explore participants’ experiences participating in a home-based exercise intervention	9 women, 12 men Treatment status: On-treatment Age range: 38–70 years Mean age: 52.0 years	Flexibility, aerobic, and resistance home-based exercises	Interviews	Observational (cross-sectional) [part of a larger experimental (randomized controlled trial)]	Qualitative/Content analysis	Cancer-related fatigue, Theory of explanatory models, Constructivist paradigm	Medium
**Prostate Cancer (*n* = 4)**									
Bruun et.al. [68]	DNK	Explore the meaning of recreational football as a team and interaction-oriented health-promoting activity	26 men Treatment status: On-treatment Age range: 58–74 years Mean age: 67.1 years	12-weeks of structured, outdoor recreational football training	Focus groups and observations	Quasi-experimental (case control) and Experimental (randomized controlled trial)	Qualitative(ethnography)/Thematic framework analysis	Not stated	Medium
Cormie et al. [69]	AUS	Describe the experience of participating in an exercise program and explore motivation for continued participation	12 men Treatment status: On- and off-treatment Age range: not stated Mean age: 75.3 years	12-weeks of structured, aerobic and resistance group-based exercise in a clinic setting	Interviews	Observational (cross-sectional)	Qualitative (phenomenology)/Content analysis	Not stated	Medium
Keogh et al. [70]	NZL	Examine perceptions of QoL and physical activity	14 men Treatment status: On- and off-treatment Age range: not stated Mean age: 65.4	Not stated	Focus groups	Observational (cross sectional)	Qualitative/Inductive thematic analysis	Not stated	Medium
Wright-St Clair et al. [71]	NZL	Explore the lived experiences of physically active men on androgen deprivation therapy	3 men Treatment status: On-treatment Age range: 74–88 years Mean age: not stated	6-months of unstructured, self-directed training	Interviews	Observational (cross sectional)	Qualitative (phenomenology)/Hermeneutic phenomenological analysis	Not stated	Medium

Notes. Med: medium; QoL: quality of life.

**Table 2:** Summary of themes and subthemes from included studies.

Theme	Subtheme	Group/Team-Based	Individual-Based	Combined Group and Indivual-Based	Sample Quotations
Physical well-being	Improved physical and functional health	[33,35,36,37,42,43,44,45,46,48,50,51,55,57,59,60,61,62,65,66,68,69]	[32,40,52,53,54,56,58,67]	[38,39,41,49,64]	“…It [walking] kept you fitter, you know, I find even going up and down stairs pretty easy whereas other people I have been talking to, that weren’t on the programme didn’t find it easy going up and down stairs and things like that. But I do definitely think the walking helped me.” [54] “I found [dragon boat racing] actually improved my physical condition. I used to have very severe osteoporosis, and I had lower back pain, and when I started paddling, because you use your whole body and you use your lower back, I was worried that it would cause too much strain on my back and it would be difficult, but it had the opposite effect. After a while, my back pain actually went away, so it was really beneficial.” [45]
	Managing the physical consequences of cancer and its treatment	[33,45,57,60,61,66,69]	[40,52,56,67]	[38,39,41]	“I just felt so good, I felt like I was in great shape and I felt energetic and I felt positive.” [45] “I have had 8 treatments, and the last 4 of them gave a lot of pain in my joints and in the rest of the body. Moving around so much has helped me cope with the pain. Being in motion helps” [40] “…But one bit that you can recover is your physical ability and strength, which exercise gives you. So that’s one thing that you can do to counter some not all of the effects of this treatment.” [70]
Psychological well-being	Focusing on health rather than illness	[33,42,44,48,50,51,55,61,69]	[32,40,53,54,67]	[39,41]	“I mean, I realize that this exercise is doing me good and probably keeping the cancer somewhat at bay...’ [69] Walking up that mountain is like taking one step away from cancer, getting away from it. It is reassuring myself that I will be better. I do not want to live in the dark any longer… It reminds me that I am moving farther away from the dark and into the light. I feel good.” [35]
					“Going through treatment, I didn’t feel healthy. I felt like my body being poisoned in order to get rid of the cancer...But the one thing—it was a mental thing—that I knew I was doing that was good for my body was exercising, and that helped me balance the process out a little better.” [33] “When I spent time together with people and could talk about other things than having cancer I that I was in a research project, and exercising. It was important to me to stop talking about the cancer and start talking about exercise. It was a nice thing to do, to be able to change the subject.” [40]
	(Re)discovering strength and physical capabilities	[34,42,43,44,45,46,47,48,50,51,55,59,62,66,68,69,70]	[40,52,53,67,71]	[41,49]	“…to be able to go to a place where I can affirm for myself that yes, my body is still capable of doing exercise, and it’s still capable of getting stronger is just enormously important psychologically. And I’m really, you know, feeling good about it because there’s something I can do to protect myself.” [62] “I really surprised myself at how much I could do. At times on the mountain I felt so strong and capable that I believed nothing could stop me from reaching my goal … If I think about it, I am a lot stronger both physically and mentally than I ever gave myself credit for. I think I can do almost anything now and that feels great.” [34]
	Exercising control and taking action	[33,35,45,47,48,50,51,55,62,63,65,68,69]	[40,52]	[41]	“It [going through cancer] is at a time in your life when you have every little control over anything else that could happen physically to you. So that to me was huge. I make the decision, “I am attending. I can do this.” It was getting that control back of my body I guess really...I don’t know how you can get that across to people?” [47]
	Evoking positive self-perceptions and minimizing negativity	[33,34,35,36,42,43,44,45,46,47,50,51,55,56,60,62,63,65,66,67,68,69,70]	[32,40,52,54,56,58]	[38,39,49]	“Psychologically, it makes you feel like you are empowered because you can do something. It [exercise] gets you out of yourself. For one, it gets you socially around people in a healthy environment. And it gets you out of just thinking about yourself and your pain and your grief and all of the emotional drama and fear of dying and anxieties...it just raised my spirits...” [33]
					“I felt the strongest I ever felt in my life. I felt a sense of confidence within myself, that was sort of based in the physical but it went beyond that. I know that the feedback that I got from people was tremendous. They were like, “What have you been doing!” It really showed...This is going to sound kind of crazy, but it brought on that Superwoman kind of feeling, like, first of all, I’ve lived through this whole trial by fire of breast cancer and I’ve come out the other end!” [33] “I think exercise helps with stress—that’s a huge one and a big factor for me. I think that had a lot to do with why I got cancer so I think it is very helpful that way.” [33]
	Gaining a sense of normalcy	[33,45,48,50,61,62]	[32,40,67]	[39,41,64]	“... During that time it’s hard to feel normal because everything has changed, but with dragon boat racing I just felt so, so normal. And from my everyday life, that was so uncomfortable for so long, for this 2 hours that I’m with them [teammates], twice a week, it was a reprieve. It was 4 hours a week that made me feel normal, 4 hours a week that I felt so good and felt a little bit like I could cope. It’s so important to feel normal [throughout breast cancer survivorship]. I think it helps you recover a lot faster and better. I think if I wasn’t doing the dragon boat racing I think I would be in really bad shape emotionally. And no matter how bad I’m feeling physically, emotionally I feel really happy.” [45]
Social well-being	Feeling understood by others	[33,35,37,42,43,44,45,46,47,48,50,55,60,61,62,63,68,69]		[41,49,64]	“... literally, we’re all in the same boat ...we all have the same feeling, like we all come from the same place. And we all understand each other ... the benefit is being with women, who the unspoken word is we’re all here together for the same reason. [48]
	Fostering social connections	[34,37,42,43,44,45,46,47,50,55,59,61,62,65,68,69]	[40,52,53,54,58]	[38,39,41,64]	“I live alone and it gets you out, gets one out, and it gets you meeting people, which is a good thing.” [65] The class is good because everyone is very open and helpful.” [38]
	Giving and receiving support	[33,35,36,37,42,43,44,45,46,47,48,50,57,60,61,62,65,68,69]	[54]	[41,64]	“We support each other, we offer practical information about how to deal with what decisions we made and it’s really comforting, to know that they have had those things, dealing with fear and the unknown of whether you are going to live” [47]
Spiritual well-being	(Re)defining life purpose and living meaningfully	[33,34,35,36,42,45,46,47,51,55,60,62,65,69]	[56,71]		“There’s a purpose, there’s a reason, and I think when you are going through what I and many others are going through, then it’s exactly what you need, a purpose. Something to get you up and out of your jammy’s in the morning. I can’t imagine not doing it, what would be the alternative? Just sitting around at home for 15 weeks waiting for the operation? It helps me feel like I am taking control and doing something to help myself.” [35] “...but something was really missing from my life. I was quite selfish. I joined it [dragon boat] to see if I could help myself, to find this missing thing.” [47]
	Becoming mindful	[34,36,45,46,48,55,57,61,66,68,69]	[56]		“Believe me, you are out there and you concentrate, you have got a lot of things you have to think about. You know, where your arms are, and your hands are, and your legs are, and your feet are, and how you are sitting and leaning out and where you are looking....” [46] “It’s like I don’t really think about the future. I don’t really think about the health. I’m so much more in the here and now. Being able to step out of the craziness of the constantly being in the future.” [36]

## Appendix

**Table A1:** MEDLINE Search Strategy.

1.	exp exercise/
2.	physical fitness/
3.	exp exercise therapy/
4.	exp sports/
5.	exp yoga/
6.	exercise test/
7.	exp physical endurance/
8.	exercise*.tw,kw.
9.	sport*.tw,kw.
10.	((physical* or strength or resistance or muscl* or muscul*) adj2 (activ* or train* or fit*4 or condition*)).tw,kw.
11.	aerobic*.tw,kw.
12.	endurance*.tw,kw.
13.	flexibility.tw,kw.
14.	stretching.tw,kw.
15.	((muscl* adj1 stretch*) or (motion adj1 therap*)).tw,kw.
16.	plyometric.tw,kw.
17.	(swimming or swim).tw,kw.
18.	(running or run).tw,kw.
19.	(walking or walk).tw,kw.
20.	or/1–19
21.	exp neoplasms/
22.	(cancer* or tumo?r* or oncolog* or leuk?emia* or carcinoma* or adeno-carcinoma* or neoplas* or lymphoma* or malignan* or melanoma* or metasta* or sarcoma* or adenoma* or adenocarcinoma* or blastoma* or mesothelioma*).tw,kw.
23.	or/21–22
24.	Qualitative Research/
25.	Focus Groups/
26.	Interview/
27.	((discourse or content or thematic or narrative or conversation) adj2 analy*).tw,kw.
28.	Ethnograph*.tw,kw.
29.	Narrative*.tw,kw.
30.	(participant adj2 observ*).tw,kw.
31.	((interpret* or interpretative*) adj2 descript*).tw,kw.
32.	interpret*.tw,kw.
33.	(life adj2 world*).tw,kw.
34.	(life adj2 story).tw,kw.
35.	(lived adj2 experienc*).tw,kw.
36.	(grounded adj2 (theor* or study or studies or research or analy*)).tw,kw.
37.	hermeneutic*.tw,kw.
38.	phenomenol*.tw,kw.
39.	theme*.tw,kw.
40.	(constant adj2 comparative).tw,kw.
41.	or/24–40
42.	Quality of Life/
43.	health status indicators/
44.	health status/
45.	“Activities of Daily Living”/
46.	(QOL or HRQOL or HRQL).tw,kw.
47.	(life adj2 qualit*).tw,kw.
48.	(life adj2 satisfaction).tw,kw.
49.	(health adj2 (status or level* or state*)).tw,kw.
50.	(daily adj2 (life adj2 function*)).tw,kw.
51.	(well-being or wellbeing or wellness).tw,kw.
52.	(well adj1 being).tw,kw.
53.	((function* or physical or cognitive* or emotion* or psycho* or social or sexual) adj2 (health* or adjust* or function* or abilit* or status)).tw,kw.
54.	social adjustment/
55.	(patient-reported adj2 outcome*).tw,kw.
56.	(self-report* or subjective*).tw,kw.
57.	or/42–56
58.	20 and 23 and 41 and 57

**Table A2:** Data Extraction Form.

**Study Details**	**Descriptions as Stated in the Report/Paper**	**Page/Para/Fig #**
*Aims/objectives*		
Country of origin		
Aim of study: What was the objective of the study? Research questions(s)?		
Notes:		
**Sample Characteristics**	**Descriptions as Stated in the Manuscript**	**Page/Para/Fig #**
*Participants*		
Sample size (include sample size for each group if more than one)		
Age (i.e., median, mean and range if possible)	Median: Mean: Range:	
Sex	M □ (*n*= ) F □ (*n*= )	
Cancer diagnosis information (i.e., type, stage, treatment status; on-/off-treatment)		
Notes:		
** Intervention Characteristics/Methods **	**Descriptions as Stated in the Manuscript**	**Page/Para/Fig #**
*Intervention*		
Exercise intervention details (i.e., setting, group based/individualized, frequency, intensity, duration, type, length of intervention)		
*Data collection*		
How was the data collected? How many qualitative data collection times were there (i.e., pre-intervention, post-intervention, follow-up)?		
How was the data analyzed?		
*Data analysis*		
Do the authors state their philosophical assumptions?	Yes □ No □ Describe:	
What guiding concept/theoretical framework was used?		
Notes:		
**Results**	**Descriptions as Stated in the Manuscript**	**Page/Para/Fig**
What is/are the main theme(s) identified?		
What are the subthemes identified?		
Raw data extracted related to theme(s) (i.e., participant quotes)		
Notes:		
